# Supplementary material for: Age and pattern of the southern high-latitude continental end-Permian extinction constrained by multiproxy analysis
Source: Nat Commun. 2019 Jan 23;10:385. doi: 10.1038/s41467-018-07934-z (PMC6344581; doi:10.1038/s41467-018-07934-z)
Supplement: Supplementary file 1 — Supplementary Information [file 41467_2018_7934_MOESM1_ESM.pdf]

Supplementary Information

Age and pattern of the southern high-latitude continental end-Permian extinction constrained by multiproxy analysis

Christopher R. Fielding[1], Tracy D. Frank[1], Stephen McLoughlin[2], Vivi Vajda[2], Chris Mays[2], Allen P. Tevyaw[1], Arne Winguth[3], Cornelia Winguth[3], Robert S. Nicoll[4], Malcolm Bocking[5], James L. Crowley[6]

[1] Department of Earth & Atmospheric Sciences, 126 Bessey Hall, University of Nebraska-Lincoln, NE 68588-0340, USA

[2] Swedish Museum of Natural History, Box 50007, S-104 05, Stockholm, Sweden

[3] Department of Earth & Environmental Sciences, University of Texas at Arlington, PO Box 19049, Arlington, TX 76019, USA

[4] Geoscience Australia, GPO Box 378, Canberra, ACT 2601, Australia

[5] Bocking Associates, 8 Tahlee Close, Castle Hill, NSW 2154, Australia

[6] Isotope Geology Laboratory, Boise State University, 1910 University Drive, Boise, ID 83725-1535, USA

Correspondence and requests for materials should be addressed to C.R.F. (email: cfielding2@unl.edu).

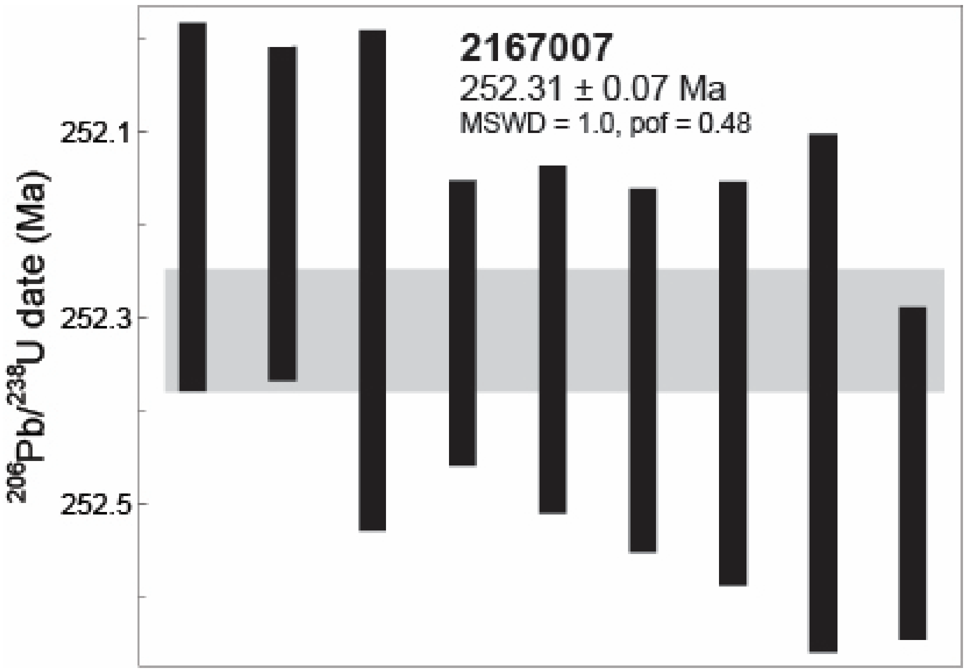

Supplementary Figure 1: Plot of $^{206}Pb/^{238}U$ dates from single grains and fragments of zircon from GA2167007 analyzed by CATIMS. Plotted with Isoplot 3.0[1]. Error bars are at the 2σ confidence interval. A weighted mean date is shown and represented by the grey boxes behind the error bars.

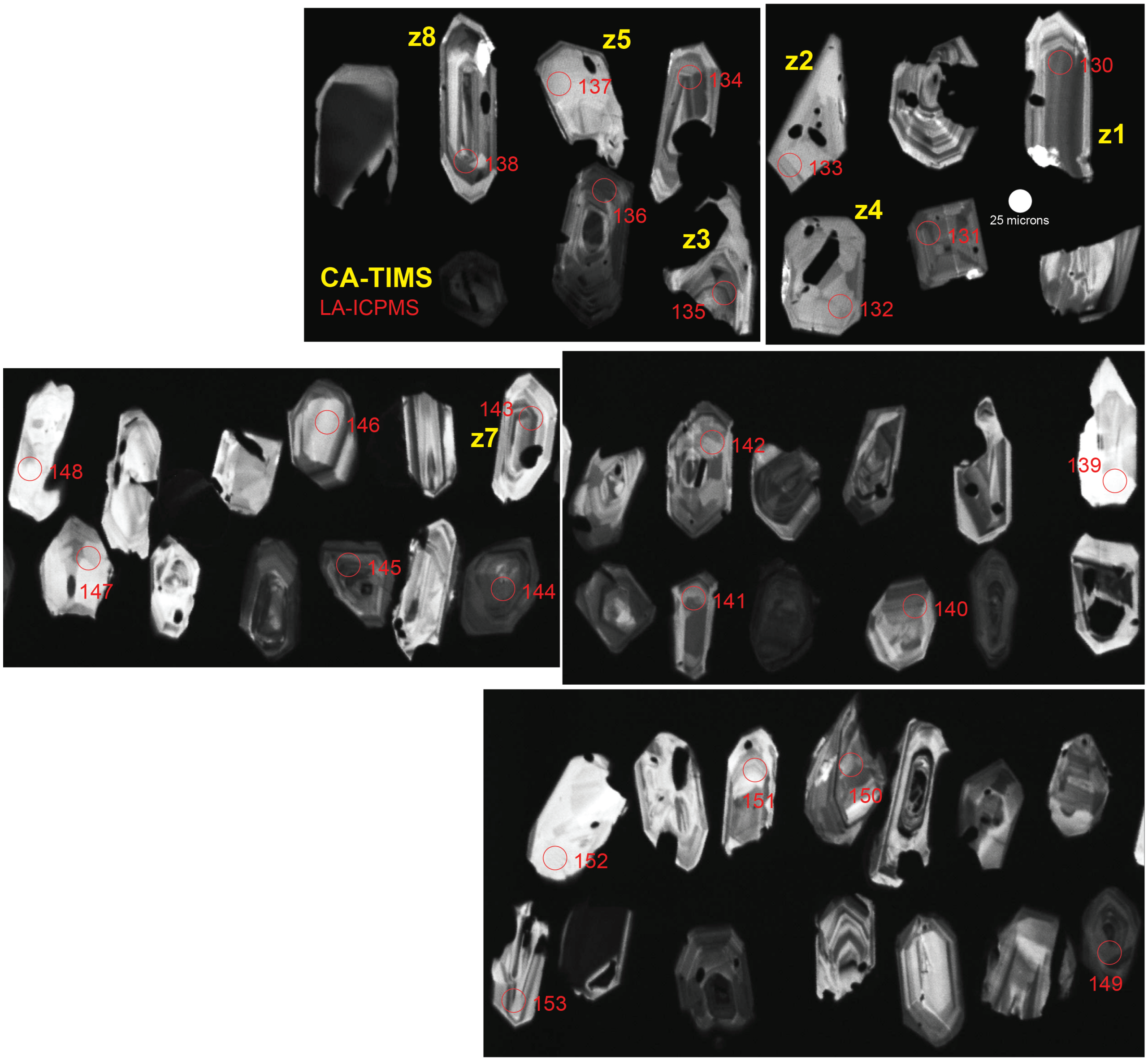

Supplementary Figure 2: Cathodoluminescence images of zircon from GA2167007. Indicated are LA-ICPMS spot locations and analysis labels and grains dated by CA-TIMS with analysis labels.

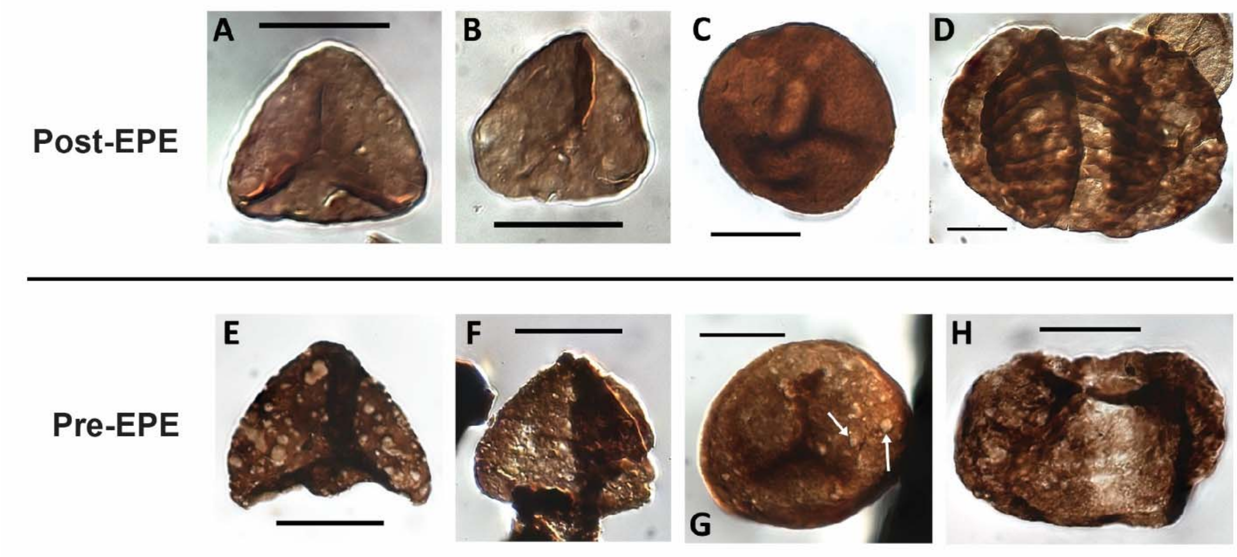

Supplementary Figure 3: Preservation styles of common terrestrial palynomorphs from PHKB1 (scale bars = 20 mm). A–D) Typical specimens from carbonaceous siltstone samples above the base of the terrestrial End-Permian Extinction (EPE); E–H) conspecific specimens from coals or carbonaceous siltstone below the base of the EPE interval. Note: the pre-EPE specimens show a characteristic degradation pattern typical of authigenic sulphide overprinting, commonly with euhedral pores (white arrows in G). A: *Leiotriletes directus*[2], proximal view (S014117/1 [R23]); B: *L. directus*, equatorial view (S014117/1 [O23(3)]); C: *Cyclogranisporites sp.* (S014132/1 [X21(3)]); D: *Protohaploxypinus limpidus*[3,4] (S014128/1 [L33(4)]; E: *L. directus*, proximal view (S014140/1 [J34(3)]); F: *L. directus*, equatorial view (S014098/1 [D40(3)]); G: *Cyclogranisporites sp.* (S014140/2 [J41(3)]); H: *P. limpidus* (S014139/2 [V47]).

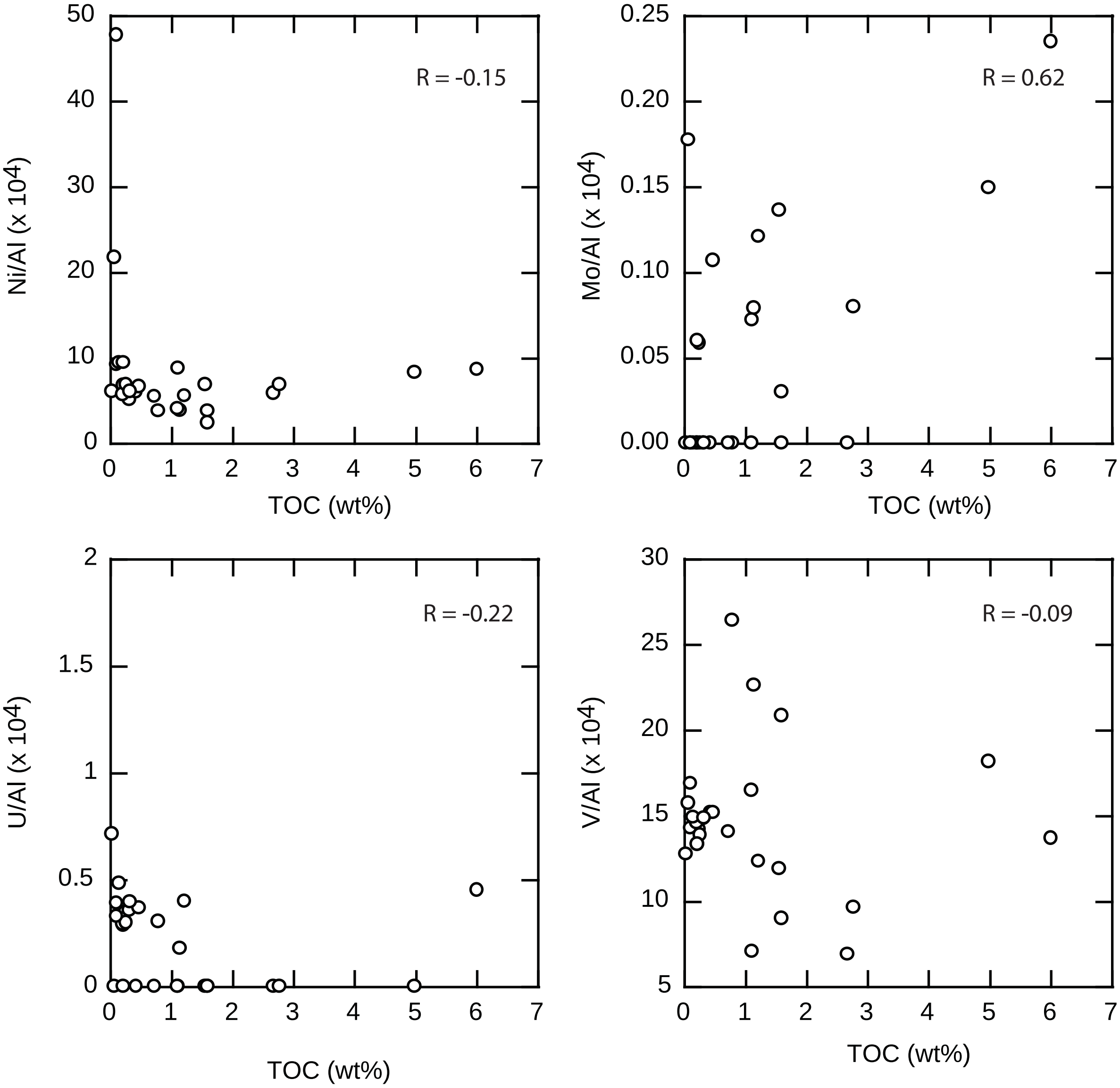

Supplementary Figure 4: Al-normalized concentrations of Ni and strongly redox-sensitive elements Mo, U, and V plotted against total organic carbon concentrations in mudrock samples from the Bunnerong-1. The correlation coefficient for each element-TOC pair is also shown. Relationships are consistent with deposition under dysoxic conditions, indicating that these trace elements resided primarily in the detrital phase. The highest Ni/Al ratios correspond to very low TOC, implicating an external source of Ni.

Supplementary Table 1. LA-ICPMS isotopic U-Pb and trace element concentration data.

Analysis	Corrected isotope ratios $^{207}Pb^*/^{235}U^*$	±2σ (%)	$^{206}Pb^*/^{238}U$	±2σ (%)	error corr.	$^{238}U/^{206}Pb^*$	±2σ (%)	$^{207}Pb^*/^{206}Pb^*$	±2σ (%)	Dates (Ma) $^{207}Pb^*/^{206}Pb^*$	±2σ (Ma)	$^{207}Pb^*/^{235}U$	±2σ (Ma)	$^{206}Pb^*/^{238}U^*$	±2σ (Ma)	% disc.
GA2167007 149	0.53513	6.40	0.06826	4.19	0.64	14.64883	4.189663711	0.05685	4.84	486	107	435	23	426	17	12
GA2167007 144	0.49841	4.63	0.06794	2.86	0.60	14.71940	2.855980942	0.05321	3.64	338	83	411	16	424	12	-25
GA2167007 146	0.49237	9.96	0.06465	4.89	0.49	15.46752	4.892557614	0.05523	8.68	422	194	407	33	404	19	4
GA2167007 147	0.38826	13.55	0.06229	6.55	0.48	16.05441	6.550291043	0.04521	11.87	-44	288	333	38	390	25	977
GA2167007 131	0.44842	7.72	0.05548	2.76	0.35	18.02417	2.759785361	0.05862	7.21	553	157	376	24	348	9	37
GA2167007 150	0.40306	8.55	0.05399	5.07	0.59	18.52320	5.068761001	0.05415	6.89	377	155	344	25	339	17	10
GA2167007 145	0.38477	6.33	0.05316	4.75	0.74	18.81239	4.750467305	0.05250	4.18	307	95	331	18	334	15	-9
GA2167007 136	0.34004	8.40	0.04659	3.87	0.45	21.46552	3.874447484	0.05294	7.46	326	169	297	22	294	11	10
GA2167007 151	0.32494	10.01	0.04304	5.36	0.53	23.23502	5.356986147	0.05476	8.46	402	189	286	25	272	14	32
GA2167007 138	0.30501	10.32	0.04231	4.36	0.42	23.63398	4.364823961	0.05228	9.35	298	213	270	24	**267**	**11**	10
GA2167007 130	0.28287	10.94	0.04113	3.73	0.33	24.31591	3.733396953	0.04989	10.28	190	239	253	24	**260**	**10**	-37
GA2167007 140	0.27010	10.13	0.04020	4.17	0.41	24.87778	4.173832846	0.04873	9.23	135	217	243	22	**254**	**10**	-88
GA2167007 143	0.36000	6.83	0.04014	4.52	0.65	24.91198	4.51580079	0.06504	5.12	776	108	312	18	**254**	**11**	67
GA2167007 132	0.26348	17.69	0.04003	5.46	0.31	24.98136	5.457575977	0.04774	16.82	86	399	237	37	**253**	**14**	-193
GA2167007 135	0.32695	9.07	0.03922	2.78	0.30	25.49748	2.781291023	0.06046	8.63	620	186	287	23	**248**	**7**	60
GA2167007 133	0.23792	15.88	0.03919	4.83	0.30	25.51491	4.833346734	0.04403	15.13	-109	372	217	31	**248**	**12**	327
GA2167007 139	0.32124	11.16	0.03917	4.94	0.44	25.52707	4.937750573	0.05947	10.01	584	217	283	28	**248**	**12**	58
GA2167007 137	0.29540	11.86	0.03849	4.38	0.36	25.97925	4.376604495	0.05566	11.02	439	245	263	27	**243**	**10**	45
														**252**	**5**	Weighted mean date based on dates shown bold. Error is 2 σ and includes standard calibration uncertainty.

Analysis	Concentrations (ppm) P	Ti	Y	Nb	La	Ce	Pr	Nd	Sm	Eu	Gd	Tb	Dy	Ho	Er	Tm	Yb	Lu	Hf	Ta	Th	U	Ti-in-zircon T(°C)
GA2167007 149	545	6.46	1895.5	1.66	0.03	6.89	0.07	2.19	5.13	0.36	33.24	12.25	167.5	64.4	292.7	62.2	543.1	110.75	9754.33	1.40	173.4	326	726
GA2167007 144	161	5.30	1117.9	1.00	0.07	17.78	0.04	2.42	4.38	1.16	21.36	7.69	91.0	35.8	164.9	36.6	352.3	80.05	10452.16	0.55	281.7	279	709
GA2167007 146	315	12.51	1177.8	0.53	0.08	2.11	0.12	2.35	5.31	0.33	33.18	9.18	107.2	39.0	181.4	33.3	295.6	61.38	9949.65	0.46	68.3	115	790
GA2167007 147	163	6.89	1028.6	0.61		4.62	0.06	1.49	3.80	0.58	22.91	7.87	92.4	35.6	152.8	31.4	269.8	56.52	8446.36	0.21	59.2	109	732
GA2167007 131	139	9.31	1255.6	0.19		11.61	0.08	2.41	6.59	1.09	26.28	9.31	115.1	41.0	189.6	39.8	380.7	64.54	8312.00	0.28	127.2	169	760
GA2167007 150	191	3.27	1207.5	3.82		28.55	0.07	0.51	3.06	0.88	20.91	7.75	93.9	39.9	189.1	43.3	415.3	88.52	12225.83	1.63	146.9	256	668
GA2167007 145	244	3.49	2253	5.37		23.76	0.14	2.22	5.66	0.83	42.49	15.13	192.0	76.1	357.0	73.4	658.4	138.72	9691.63	1.30	346.3	467	674
GA2167007 136	159	2.09	915.31	1.50		8.55		0.89	1.12	0.12	12.90	4.66	71.5	30.0	148.2	35.7	345.4	64.98	10301.59	0.81	134.0	275	634
GA2167007 151	440	3.78	2260.6	3.34	0.04	17.72	0.01	0.92	3.44	0.91	27.47	11.34	171.4	72.6	366.7	84.3	811.8	184.05	10705.96	1.17	113.6	206	680
GA2167007 138	393	3.80	1787.1	2.42		13.06	0.02	0.55	2.77	0.87	25.29	9.87	132.0	58.2	295.1	71.1	740.3	142.50	8741.99	0.85	66.7	138	680
GA2167007 130	409	6.67	2493.9	1.56		15.84	0.12	2.61	6.61	2.41	44.01	16.78	212.5	86.2	400.7	95.7	932.7	171.78	7513.76	0.64	133.0	196	729
GA2167007 140	138	8.01	636.61	0.66		5.71		0.78	1.20	0.26	11.47	3.92	51.9	20.3	99.2	20.5	200.5	41.84	9349.86	0.25	102.7	174	746
GA2167007 143	503	4.37	2841.6	4.79		27.23	0.01	1.08	3.67	1.50	39.99	16.19	225.7	92.3	451.5	99.4	942.4	210.37	10740.82	1.55	275.6	357	692
GA2167007 132	293	6.39	1573	1.71	0.93	7.93	0.62	2.71	4.37	0.80	30.36	11.76	145.9	56.6	256.1	55.0	522.6	86.19	6958.44	0.74	46.2	101	725
GA2167007 135	614	2.91	2702.8	4.63		23.93		0.82	5.20	1.04	37.92	15.93	220.6	91.8	439.5	105.8	1046.3	192.27	9761.50	1.70	266.4	410	659
GA2167007 133	230	7.46	1092.5	1.37		4.94	0.03	1.05	3.50	0.60	20.19	7.89	99.8	39.7	174.6	38.0	361.5	63.00	6989.83	0.62	40.9	89	739
GA2167007 139	454	11.44	2924.5	2.23	0.01	21.40	0.11	2.09	5.12	2.73	43.96	16.83	229.4	100.3	474.4	101.5	977.3	213.50	8381.44	0.55	59.9	79	781
GA2167007 137	359	5.98	1122	1.45	0.89	9.50	0.40	2.66	3.73	0.49	23.65	8.62	101.1	39.5	180.0	40.8	387.1	65.75	7987.90	0.59	43.8	91	719

Experiment on January 31, 2018

Isotope ratio and date errors include systematic calibration errors of 0.72% ($^{207}Pb/^{206}Pb$) and 1.22% ($^{206}Pb/^{238}U$) (2 σ).

Ablation used a laser spot size of 25 μm and a laser firing repetition rate of 10 Hz.

Activity of TiO_2 for Ti-in-Zircon temperature calculation is 0.8.

Supplementary Table 2. U-Pb isotopic data.

							Radiogenic Isotope Ratios								Isotopic Dates					
Sample (a)	Th/U (b)	^{206}Pb* x10^{-13} mol (c)	mol % ^{206}Pb* (c)	Pb*/Pb$_c$ (c)	Pb$_c$ (pg) (c)	^{206}Pb/^{204}Pb (d)	^{208}Pb/^{206}Pb (e)	^{207}Pb/^{206}Pb (e)	% err (f)	^{207}Pb/^{235}U (e)	% err (f)	^{206}Pb/^{238}U (e)	% err (f)	corr. coef.	^{207}Pb/^{206}Pb (g)	± (f)	^{207}Pb/^{235}U (g)	± (f)	^{206}Pb/^{238}U (g)	± (f)
**GA2167007**																				
**z1a**	0.513	0.2055	98.94%	28	0.18	1707	0.163	0.051236	0.261	0.281995	0.306	0.039918	0.076	0.683	251.45	5.99	252.24	0.68	252.32	0.19
**z1b**	0.559	0.0964	96.98%	10	0.25	598	0.177	0.051047	0.925	0.280884	0.999	0.039908	0.109	0.701	242.96	21.32	251.36	2.22	252.26	0.27
**z2a**	0.565	0.2120	98.86%	26	0.20	1579	0.179	0.051316	0.329	0.282474	0.371	0.039923	0.079	0.616	255.05	7.56	252.62	0.83	252.36	0.20
**z2b**	0.634	0.1787	98.41%	19	0.24	1136	0.201	0.051181	0.415	0.281672	0.471	0.039915	0.062	0.926	248.99	9.54	251.98	1.05	252.30	0.15
**z3**	0.532	0.2815	98.65%	22	0.32	1332	0.169	0.051210	0.353	0.282015	0.399	0.039941	0.072	0.690	250.29	8.12	252.26	0.89	252.47	0.18
**z4a**	0.521	0.1905	98.18%	16	0.29	989	0.165	0.051386	0.491	0.282877	0.541	0.039925	0.088	0.630	258.20	11.27	252.94	1.21	252.37	0.22
**z5**	0.570	0.2300	98.20%	17	0.35	1003	0.181	0.051230	0.424	0.281797	0.471	0.039895	0.080	0.652	251.17	9.74	252.08	1.05	252.18	0.20
**z7**	0.511	0.1280	98.46%	19	0.17	1171	0.162	0.051255	0.484	0.282166	0.536	0.039927	0.112	0.550	252.31	11.13	252.37	1.20	252.38	0.28
**z8**	0.479	0.3400	99.45%	54	0.16	3264	0.152	0.051242	0.174	0.281876	0.218	0.039896	0.073	0.718	251.74	4.00	252.14	0.49	252.19	0.18

(a) Labels for analyses composed of single zircon grains or fragments that were annealed and chemically abraded[5]. Fragments from same grain are denoted by a and b.

(b) Model Th/U ratio calculated from radiogenic ^{208}Pb/^{206}Pb ratio and ^{207}Pb/^{235}U date.

(c) Pb* and Pbc are radiogenic and common Pb, respectively. mol % ^{206}Pb* is with respect to radiogenic and blank Pb.

(d) Measured ratio corrected for spike and fractionation only. Fractionation correction is 0.16 ± 0.03 (1 σ) %/amu (atomic mass unit) for single-collector Daly analyses, based on analysis of EARTHTIME ^{202}Pb-^{205}Pb tracer solution.

(e) Corrected for fractionation and spike. Common Pb in zircon analyses is assigned to procedural blank with composition of ^{206}Pb/^{204}Pb = 18.04 ± 0.61%; ^{207}Pb/^{204}Pb = 15.54 ± 0.52%; ^{208}Pb/^{204}Pb = 37.69 ± 0.63% (1 sigma). ^{206}Pb/^{238}U and ^{207}Pb/^{206}Pb ratios corrected for initial disequilibrium in ^{230}Th/^{238}U using D[Th/U] = 0.20 ± 0.05 (1 σ).

(f) Errors are 2 sigma, propagated using published algorithms[6,7].

(g) Calculations based on the published decay constants[8]. ^{206}Pb/^{238}U and ^{207}Pb/^{206}Pb dates corrected for initial disequilibrium in ^{230}Th/^{238}U using D[Th/U] = 0.20 ± 0.05 (1 σ).

Supplementary Table 3: Palynofacies from Bunnerong-1 (PKHB1)

Depth (m)	Sample #	Strati-graphic height from top of Bulli Coal seam (m)	Palynofacies category																														Total count
			Palynomorphs															Phytoclasts											AOM				
			Plant spores				Pollen			Phytoplankton			Fungal debris			Subtotal (n)	Subtotal (%)	Opaque			Other trans-lucent	Tracheids & rays	Cuticles & mem-branes	All brown phytoclasts		Opaque/ brown	Subtotal (n)	Subtotal (%)	Parti-culate (≥5μm)	Resin	Subtotal (n)	Subtotal (%)	
			n	% total	% palyn	% mio-spores	n	% total	% palyn	n	% total	% palyn	n	% total	% paly			n	% total	% phyto				% total	% phyto								
239.75	S014137	565.33	28	5.6	63.64	73.68	10	2.0	22.73	6	1.2	13.64	0	0.0	0.00	44	8.80	17	3.4	4.11	216	119	62	79.4	95.89	0.04	414	82.80	40	2	42	8.40	500
267.55	S014136	537.53	26	5.2	65.00	70.27	11	2.2	27.50	3	0.6	7.50	0	0.0	0.00	40	8.00	25	5.0	5.68	182	149	84	83.0	94.32	0.06	440	88.00	20	0	20	4.00	500
290.74	S014135	514.34	19	3.8	57.58	65.52	10	2.0	30.30	4	0.8	12.12	0	0.0	0.00	33	6.60	18	3.6	4.12	220	181	18	83.8	95.88	0.04	437	87.40	30	0	30	6.00	500
320.1	S014134	484.98	144	28.8	84.71	88.34	19	3.8	11.18	7	1.4	4.12	0	0.0	0.00	170	34.00	12	2.4	3.86	85	144	70	59.8	96.14	0.04	311	62.20	19	0	19	3.80	500
354	S014133	451.08	39	7.8	63.93	65.00	21	4.2	34.43	1	0.2	1.64	0	0.0	0.00	61	12.20	37	7.4	8.87	187	142	51	76.0	91.13	0.10	417	83.40	22	0	22	4.40	500
362	S014132	443.08	45	9.0	64.29	65.22	24	4.8	34.29	1	0.2	1.43	0	0.0	0.00	70	14.00	57	11.4	13.57	184	162	17	72.6	86.43	0.16	420	84.00	10	0	10	2.00	500
383.48	S014131	421.60	66	13.2	64.71	69.47	29	5.8	28.43	5	1.0	4.90	2	0.4	1.96	102	20.40	5	1.0	1.42	93	164	89	69.2	98.58	0.01	351	70.20	47	0	47	9.40	500
439.95	S014129	365.13	28	5.6	62.22	73.68	10	2.0	22.22	7	1.4	15.56	0	0.0	0.00	45	9.00	9	1.8	2.91	68	19	213	60.0	97.09	0.03	309	61.80	146	0	146	29.20	500
477.2	S014128	327.88	41	8.2	64.06	70.69	17	3.4	26.56	6	1.2	9.38	0	0.0	0.00	64	12.80	9	1.8	2.78	93	48	174	63.0	97.22	0.03	324	64.80	112	0	112	22.40	500
514.85	S014127	290.23	71	14.2	71.00	83.53	14	2.8	14.00	12	2.4	12.00	3	0.6	3.00	100	20.00	13	2.6	4.06	140	95	72	61.4	95.94	0.04	320	64.00	80	0	80	16.00	500
559.6	S014126	245.48	29	5.8	54.72	65.91	15	3.0	28.30	9	1.8	16.98	0	0.0	0.00	53	10.60	29	5.8	8.95	156	105	34	59.0	91.05	0.10	324	64.80	123	0	123	24.60	500
587.47	S014125	217.61	47	9.4	57.32	63.51	27	5.4	32.93	8	1.6	9.76	0	0.0	0.00	82	16.40	22	4.4	5.61	166	169	35	74.0	94.39	0.06	392	78.40	26	0	26	5.20	500
614.33	S014124	190.75	18	3.6	52.94	56.25	14	2.8	41.18	2	0.4	5.88	0	0.0	0.00	34	6.80	27	5.4	6.19	234	166	9	81.8	93.81	0.07	436	87.20	29	1	30	6.00	500
637.36	S014123	167.72	5	1.0	55.56	83.33	1	0.2	11.11	1	0.2	11.11	2	0.4	22.22	9	1.80	10	2.0	4.48	182	26	5	42.6	95.52	0.05	223	44.60	268	0	268	53.60	500
661.09	S014122	143.99	4	0.8	36.36	50.00	4	0.8	36.36	3	0.6	27.27	0	0.0	0.00	11	2.20	30	6.0	11.03	218	19	5	48.4	88.97	0.12	272	54.40	217	0	217	43.40	500
675.68	S014121	129.40	12	2.4	54.55	57.14	9	1.8	40.91	1	0.2	4.55	0	0.0	0.00	22	4.40	17	3.4	6.37	224	23	3	50.0	93.63	0.07	267	53.40	210	1	211	42.20	500
699.07	S014120	106.01	21	4.2	46.67	48.84	22	4.4	48.89	2	0.4	4.44	0	0.0	0.00	45	9.00	26	5.2	6.47	202	167	7	75.2	93.53	0.07	402	80.40	53	0	53	10.60	500
709.24	S014119	95.84	3	0.6	50.00	60.00	2	0.4	33.33	1	0.2	16.67	0	0.0	0.00	6	1.20	56	11.2	11.55	312	116	1	85.8	88.45	0.13	485	97.00	9	0	9	1.80	500
728.11	S014118	76.97	7	1.4	41.18	50.00	7	1.4	41.18	3	0.6	17.65	0	0.0	0.00	17	3.40	15	3.0	3.18	260	167	30	91.4	96.82	0.03	472	94.40	10	1	11	2.20	500
745.62	S014117	59.46	34	6.8	50.75	56.67	26	5.2	38.81	7	1.4	10.45	0	0.0	0.00	67	13.40	23	4.6	5.99	193	160	8	72.2	94.01	0.06	384	76.80	49	0	49	9.80	500
760.09	S014116	44.99	9	1.8	52.94	56.25	7	1.4	41.18	1	0.2	5.88	0	0.0	0.00	17	3.40	47	9.4	10.02	306	111	5	84.4	89.98	0.11	469	93.80	14	0	14	2.80	500
773.96	S014115	31.12	17	3.4	45.95	60.71	11	2.2	29.73	9	1.8	24.32	0	0.0	0.00	37	7.40	12	2.4	3.23	271	72	17	72.0	96.77	0.03	372	74.40	91	0	91	18.20	500
778.57	S014114	26.51	22	4.4	34.38	36.07	39	7.8	60.94	3	0.6	4.69	0	0.0	0.00	64	12.80	43	8.6	11.08	228	106	11	69.0	88.92	0.12	388	77.60	48	0	48	9.60	500
781.18	S014113	23.90	14	2.8	41.18	50.00	14	2.8	41.18	6	1.2	17.65	0	0.0	0.00	34	6.80	21	4.2	7.69	190	61	1	50.4	92.31	0.08	273	54.60	193	0	193	38.60	500
781.95	S014149	23.13	6	1.2	42.86	54.55	5	1.0	35.71	3	0.6	21.43	0	0.0	0.00	14	2.80	58	11.6	13.98	306	41	10	71.4	86.02	0.16	415	83.00	69	2	71	14.20	500
782.27	S014112	22.81	10	2.0	58.82	62.50	6	1.2	35.29	1	0.2	5.88	0	0.0	0.00	17	3.40	46	9.2	10.82	327	51	1	75.8	89.18	0.12	425	85.00	58	0	58	11.60	500
783.21	S014111	21.87	11	2.2	55.00	61.11	7	1.4	35.00	2	0.4	10.00	0	0.0	0.00	20	4.00	34	6.8	7.51	354	62	3	83.8	92.49	0.08	453	90.60	27	0	27	5.40	500
783.45	S014148	21.63	35	7.0	46.05	51.47	33	6.6	43.42	8	1.6	10.53	0	0.0	0.00	76	15.20	37	7.4	9.59	262	82	5	69.8	90.41	0.11	386	77.20	38	0	38	7.60	500
786.37	S014108	18.71	21	4.2	48.84	61.76	13	2.6	30.23	9	1.8	20.93	0	0.0	0.00	43	8.60	40	8.0	8.97	321	79	6	81.2	91.03	0.10	446	89.20	11	0	11	2.20	500
786.65	S014147	18.43	15	3.0	45.45	51.72	14	2.8	42.42	4	0.8	12.12	0	0.0	0.00	33	6.60	52	10.4	11.40	321	79	4	80.8	88.60	0.13	456	91.20	10	1	11	2.20	500
787.52	S014107	17.56	27	5.4	45.00	48.21	29	5.8	48.33	4	0.8	6.67	0	0.0	0.00	60	12.00	37	7.4	8.79	340	41	3	76.8	91.21	0.10	421	84.20	18	1	19	3.80	500
788.2	S014106	16.88	31	6.2	43.66	53.45	27	5.4	38.03	13	2.6	18.31	0	0.0	0.00	71	14.20	67	13.4	16.83	275	50	6	66.2	83.17	0.20	398	79.60	31	0	31	6.20	500
792.85	S014105	12.23	12	2.4	70.59	75.00	4	0.8	23.53	1	0.2	5.88	0	0.0	0.00	17	3.40	80	16.0	17.47	310	63	5	75.6	82.53	0.21	458	91.60	25	0	25	5.00	500
798.85	S014146	6.23	31	6.2	55.36	75.61	10	2.0	17.86	15	3.0	26.79	0	0.0	0.00	56	11.20	82	16.4	33.20	103	48	14	33.0	66.80	0.50	247	49.40	196	1	197	39.40	500
799.56	S014104	5.52	24	4.8	53.33	70.59	10	2.0	22.22	11	2.2	24.44	0	0.0	0.00	45	9.00	82	16.4	30.37	154	27	7	37.6	69.63	0.44	270	54.00	185	0	185	37.00	500
801.11	S014103	3.97	21	4.2	65.63	77.78	6	1.2	18.75	5	1.0	15.63	0	0.0	0.00	32	6.40	19	3.8	7.72	159	63	5	45.4	92.28	0.08	246	49.20	222	0	222	44.40	500
802.97	S014102	2.11	12	2.4	54.55	92.31	1	0.2	4.55	9	1.8	40.91	0	0.0	0.00	22	4.40	27	5.4	15.79	115	29	0	28.8	84.21	0.19	171	34.20	307	0	307	61.40	500
804.08	S014145	1.00	19	3.8	61.29	61.29	12	2.4	38.71	0	0.0	0.00	0	0.0	0.00	31	6.20	85	17.0	18.40	319	51	7	75.4	81.60	0.23	462	92.40	6	1	7	1.40	500
805.03	S014101	0.05	25	5.0	60.98	60.98	16	3.2	39.02	0	0.0	0.00	0	0.0	0.00	41	8.20	74	14.8	16.37	311	63	4	75.6	83.63	0.20	452	90.40	7	0	7	1.40	500
**Putative base of extinction interval**																																	
810.09	S014100	-5.01	10	2.0	58.82	58.82	7	1.4	41.18	0	0.0	0.00	0	0.0	0.00	17	3.40	126	25.2	26.09	318	39	0	71.4	73.91	0.35	483	96.60	0	0	0	0.00	500
810.92	S014144	-5.84	20	4.0	58.82	58.82	14	2.8	41.18	0	0.0	0.00	0	0.0	0.00	34	6.80	88	17.6	19.30	321	42	5	73.6	80.70	0.24	456	91.20	10	0	10	2.00	500
811.08	S014143	-6.00	38	7.6	70.37	70.37	16	3.2	29.63	0	0.0	0.00	0	0.0	0.00	54	10.80	55	11.0	12.94	305	58	7	74.0	87.06	0.15	425	85.00	21	0	21	4.20	500
811.67	S014142	-6.59	22	4.4	66.67	66.67	11	2.2	33.33	0	0.0	0.00	0	0.0	0.00	33	6.60	77	15.4	16.49	334	53	3	78.0	83.51	0.20	467	93.40	0	0	0	0.00	500
814.96	S014141	-9.88	13	2.6	72.22	72.22	5	1.0	27.78	0	0.0	0.00	0	0.0	0.00	18	3.60	100	20.0	20.75	329	45	8	76.4	79.25	0.26	482	96.40	0	0	0	0.00	500
815.94	S014140	-10.86	46	9.2	62.16	63.01	27	5.4	36.49	1	0.2	1.35	0	0.0	0.00	74	14.80	68	13.6	16.31	279	58	12	69.8	83.69	0.19	417	83.40	9	0	9	1.80	500
819.48	S014099	-14.40	19	3.8	67.86	67.86	9	1.8	32.14	0	0.0	0.00	0	0.0	0.00	28	5.60	96	19.2	20.47	306	66	1	74.6	79.53	0.26	469	93.80	3	0	3	0.60	500
820.64	S014139	-15.56	18	3.6	78.26	81.82	4	0.8	17.39	0	0.0	0.00	1	0.2	4.35	23	4.60	58	11.6	12.31	377	29	7	82.6	87.69	0.14	471	94.20	6	0	6	1.20	500
822.7	S014138	-17.62	55	11.0	74.32	74.32	19	3.8	25.68	0	0.0	0.00	0	0.0	0.00	74	14.80	48	9.6	11.54	297	56	15	73.6	88.46	0.13	416	83.20	10	0	10	2.00	500
840.32	S014098	-35.24	30	6.0	63.83	63.83	17	3.4	36.17	0	0.0	0.00	0	0.0	0.00	47	9.40	51	10.2	12.14	303	51	15	73.8	87.86	0.14	420	84.00	33	0	33	6.60	500
860.37	S014097	-55.29	18	3.6	52.94	52.94	16	3.2	47.06	0	0.0	0.00	0	0.0	0.00	34	6.80	40	7.8	8.69	331	62	16	82.0	91.31	0.10	449	89.80	17	0	17	3.40	500
880.32	S014096	-75.24	19	3.8	42.22	42.22	26	5.2	57.78	0	0.0	0.00	0	0.0	0.00	45	9.00	45	9.0	10.44	307	64	15	77.2	89.56	0.12	431	86.20	24	0	24	4.80	500
900.22	S014095	-95.14	47	9.4	71.21	72.31	18	3.6	27.27	0	0.0	0.00	1	0.2	1.52	66	13.20	46	9.2	12.37	259	60	7	65.2	87.63	0.14	372	74.40	62	0	62	12.40	500
		**Totals**	**1404**				**745**			**194**			**9**			**2352**		**2297**			**12653**	**4203**	**1212**				**20366**		**3271**	**11**	**3282**		**26000**

Legend

Aratrisporites tenuispinosus Zone
Protohaploxypinus samoilovichii Zone
Lunatisporites pellucidus Zone
Protohaploxypinus microcorpus Zone
Playfordiaspora crenulata Zone
Dulhuntyispora parthivola Zone

Supplementary Table 4: Spore-pollen index taxa and selected palynomorph group abundances from Bunnerong-1 (PKHB1).

			Spore-pollen index taxa										Selected palynomorph groups						
**Depth (m)**	**Sample #**	**Stratigraphic height from top of Bulli Coal seam (m)**	***Brevitriletes bulliensis*** (9 ex 10),11	***Triplexi-sporites play-fordii*** (12),13	***Triquitrites proratus*** 14	***Playfordia-spora crenulata*** (15),13	***Lundbladi-spora springsur-ensis*** 10	***Limatula-sporites* spp.**	***Rewani-spora foveolata*** 16	***Lunati-sporites pellucidus*** (17),9,18	***Aratri-sporites* spp.**	***Lundblad i-spora* sp. A**13	**Taeniate bisaccate pollen**	**Non-taeniate bisaccate pollen**	**Azonate trilete microspores**	**Zonate trilete microspores**	**Monolete spores**	**Other palynomorphs**	**Total palynomorph count**
239.75	S014137	565.33	4	2	0	0	0	1	0	0	3	0	2	68	145	13	3	19	250
267.55	S014136	537.53	4	0	0	2	0	1	0	1	1	0	6	50	154	21	1	18	250
290.74	S014135	514.34	0	0	0	0	0	0	0	0	16	0	8	47	140	24	16	15	250
320.10	S014134	484.98	0	0	0	0	0	0	0	0	157	0	3	13	41	27	157	9	250
354.00	S014133	451.08	0	0	0	0	0	0	0	3	8	0	10	44	173	6	8	9	250
362.00	S014132	443.08	1	1	0	3	0	9	0	18	12	0	40	108	95	13	20	11	287
383.48	S014131	421.60	0	0	0	0	0	0	0	0	82	0	5	33	48	53	83	28	250
439.95	S014129	365.13	1	0	0	2	0	9	0	18	6	0	37	12	102	211	7	19	388
477.20	S014128	327.88	2	2	0	0	0	2	1	28	5	0	65	15	177	305	6	14	582
514.85	S014127	290.23	2	2	0	1	1	6	0	20	16	1	59	8	201	150	16	26	460
559.60	S014126	245.48	3	2	0	0	1	9	1	18	0	0	48	32	226	51	10	33	400
587.47	S014125	217.61	17	0	0	2	1	32	15	8	0	0	40	15	285	51	30	27	448
614.33	S014124	190.75	1	0	0	0	0	0	0	1	0	0	21	11	165	36	0	17	250
637.36	S014123	167.72																	
661.09	S014122	143.99																	
675.68	S014121	129.40																	
699.07	S014120	106.01	8	1	0	1	9	11	2	4	2	0	20	108	286	16	42	42	514
709.24	S014119	95.84	2	0	0	0	0	0	0	1	16	0	11	21	149	22	18	29	250
728.11	S014118	76.97	1	0	0	0	4	0	0	1	1	0	7	23	163	22	8	27	250
745.62	S014117	59.46	4	0	0	0	1	3	1	1	2	0	30	49	139	3	8	30	259
760.09	S014116	44.99	2	0	0	0	0	2	0	0	0	0	10	11	105	20	2	19	167
773.96	S014115	31.12																	
778.57	S014114	26.51	21	1	0	0	0	0	0	0	0	0	15	166	226	2	3	24	436
781.18	S014113	23.90	1	0	2	1	2	0	0	0	0	0	16	58	139	21	1	15	250
781.95	S014149	23.13																	
782.27	S014112	22.81																	
783.21	S014111	21.87																	
783.45	S014148	21.63	4	1	0	0	0	0	0	0	0	0	8	2	167	18	0	55	250
786.37	S014108	18.71	3	0	0	0	0	0	0	0	0	0	8	2	178	7	1	54	250
786.65	S014147	18.43	3	0	0	0	0	0	0	0	0	0	13	16	163	14	2	42	250
787.52	S014107	17.56																	
788.20	S014106	16.88	2	0	0	0	0	0	0	0	0	0	6	14	154	9	3	64	250
792.85	S014105	12.23	0	0	0	0	0	0	0	0	0	0	5	5	54	5	1	3	73
798.85	S014146	6.23	6	0	0	0	0	0	0	0	0	0	6	18	146	21	1	58	250
799.56	S014104	5.52	7	0	0	0	0	0	0	0	0	0	13	29	147	24	4	33	250
801.11	S014103	3.97	17	1	0	0	0	0	0	0	0	0	11	14	229	2	24	37	317
802.97	S014102	2.11	12	2	0	0	0	0	0	0	0	0	11	5	141	11	2	80	250
804.08	S014145	1.00	3	0	0	0	0	0	0	0	0	0	11	6	216	12	4	1	250
805.03	S014101	0.05	5	3	0	0	0	0	0	0	0	0	4	1	198	7	37	3	250
**Putative base of extinction interval**																			
810.09	S014100	-5.01	0	0	0	0	0	0	0	0	0	0	8	4	135	15	5	3	170
810.92	S014144	-5.84	0	0	0	0	0	0	0	0	0	0	23	11	199	12	0	5	250
811.08	S014143	-6.00	0	0	0	0	0	0	0	0	0	0	14	12	200	16	0	8	250
811.67	S014142	-6.59	0	0	0	0	0	0	0	0	0	0	22	12	196	13	0	7	250
814.96	S014141	-9.88	0	0	0	0	0	0	0	0	0	0	13	8	65	15	0	3	104
815.94	S014140	-10.86	0	0	0	0	0	0	0	0	0	0	13	10	150	66	0	11	250
819.48	S014099	-14.40	0	0	0	0	0	0	0	0	0	0	2	0	71	3	0	0	76
820.64	S014139	-15.56	0	0	0	0	0	0	0	0	0	0	23	17	189	13	0	8	250
822.70	S014138	-17.62	0	0	0	0	0	0	0	0	0	0	42	14	179	11	0	4	250
840.32	S014098	-35.24	0	0	0	0	0	0	0	0	0	0	15	5	221	6	0	3	250
860.37	S014097	-55.29	0	0	0	0	0	0	0	0	0	0	12	5	114	9	0	3	143
880.32	S014096	-75.24	0	0	0	0	0	0	0	0	0	0	36	29	173	7	0	5	250
900.22	S014095	-95.14	0	0	0	0	0	0	0	0	0	0	6	23	204	13	0	4	250
		**Totals**	136	**18**	**2**	**12**	**19**	**85**	**20**	**122**	**327**	**1**	**778**	**1154**	**7048**	**1396**	**523**	**925**	**11824**

Blank cells indicate inadequate palynomorph recovery

Legend

Aratrisporites tenuispinosus Zone

Protohaploxypinus samoilovichii Zone

Lunatisporites pellucidus Zone

Protohaploxypinus microcorpus Zone

Playfordiaspora crenulata Zone

Dulhuntyispora parthivola Zone

Supplementary Table 5: Geochemical data from Bunnerong-1 (PKHB1)

Depth (m)	Stratigraphic height from top of Bulli Coal seam (m)	δ^{13}Corg (‰ VPDB)	CIA	Kaolinite/ Illite	TOC (wt%)	Ni/Al ($x10^4$)	Mo/Al ($x10^4$)	U/Al ($x10^4$)	V/Al ($x10^4$)
239.75	565.33	-20.3			1.4				
290.74	514.34	-21.7			0.9				
320.10	484.98	-25.6	96.8		1.6	3.81	0.03	0.00	20.83
354.00	451.08	-22.7			0.4				
362.00	443.08	-22.2	95.5		0.8	3.77	0.00	0.31	26.41
375.68	429.40	-27.9			0.0				
383.48	421.60		95.7	0.8		3.83	0.08	0.18	22.62
413.72	391.36	-24.6			0.7				
439.95	365.13	-28.0	75.7		0.3	6.14	0.06	0.35	14.20
439.95	365.13	-28.1			0.3				
477.20	327.88	-27.3			0.2				
514.85	290.23	-26.4			0.2				
559.60	245.48	-24.5			0.2				
559.60	245.48	-24.2			0.2				
587.47	217.61	-25.3	73.0	1.1	0.2	6.83	0.00	0.29	13.33
614.33	190.75	-24.6			0.1				
614.33	190.75	-24.4			0.1				
637.36	167.72	-28.2			0.0				
661.09	143.99	-22.1	76.6		0.0	6.13	0.00	0.71	12.73
675.68	129.40	-22.4			0.0				
699.07	106.01	-27.6			0.2				
713.76	91.32	-24.7			0.1				
728.11	76.97	-29.6			1.3				
733.13	71.95	-26.3			0.1				
745.62	59.46	-26.3	74.9		0.3	5.15	0.00	0.36	14.84
760.09	44.99	-29.4			0.3				
773.96	31.12	-28.7			0.1				
778.57	26.51	-27.0			0.4				
778.57	26.51								
781.18	23.90	-26.9			0.2				
781.95	23.13	-22.3			0.1				
782.27	22.81		72.2	0.9		16.62	0.00	0.64	18.22
783.21	21.87	-26.9			0.1				
783.45	21.63	-26.5			0.2				
783.81	21.27	-24.7			0.1				
784.40	20.68	-26.7	76.4		0.1	9.18	0.00	0.39	14.26
786.37	18.71	-26.2	80.6		0.2	5.77	0.00	0.30	14.57
786.65	18.43	-26.5	79.5		0.3	6.90	0.00	0.30	13.86
787.52	17.56		70.0			9.08	0.00	1.79	19.24
788.20	16.88	-22.9	76.0		0.1	9.39	0.00	0.48	14.90
792.85	12.23	-27.5	74.8		0.1	47.80	0.00	0.33	16.88
799.56	5.52	-27.4	78.6		0.1	21.77	0.18	0.00	15.74

Geochemical data, cont'd

Depth (m)	Depth (m from top of Bulli Coal seam)	δ^{13}Corg (‰ VPDB)	CIA	Kaolinite/ Illite	TOC (wt%)	Ni/Al (x10^4)	Mo/Al (x10^4)	U/Al (x10^4)	V/Al (x10^4)
801.11	3.97	-23.0	80.2		0.4	6.00	0.00	0.00	15.17
802.97	2.11	-25.3	84.0	1.8	0.7	5.44	0.00	0.00	14.04
804.08	1.00	-26.3	80.0		0.5	6.65	0.11	0.37	15.17
805.03	0.05	-26.6	79.7		0.3	6.07	0.00	0.39	14.84
810.09	-5.01	-24.2	85.7		2.7	5.88	0.00	0.00	6.90
810.92	-5.84	-23.9	83.1		1.1	8.81	0.07	0.00	7.05
811.67	-6.59	-24.1			5.3				
814.96	-9.88	-24.0			3.1				
819.48	-14.40	-23.9	74.0		1.1	4.02	0.00	0.00	16.48
820.64	-15.56	-25.0	70.9		0.2	9.39	0.06	0.00	13.34
822.70	-17.62	-24.8			0.9				
831.43	-26.35	-22.0	70.5		6.0	8.66	0.23	0.45	13.69
849.10	-44.02	-25.2			2.8				
860.37	-55.29	-24.4	68.2	0.5	1.6	6.87	0.14	0.00	11.89
869.89	-64.81	-24.0			6.8				
880.32	-75.24	-23.7	71.0		1.6	2.42	0.00	0.00	9.00
883.30	-78.22	-24.8			2.3				
900.25	-95.17	-23.9			4.4				
902.38	-97.30	-25.3			7.1				
909.05	-103.97	-23.7			2.6				
910.92	-105.84	-24.5			1.1				
940.75	-135.67	-22.2	68.5		2.8	6.87	0.08	0.00	9.64
952.90	-147.82	-25.0			1.2				
996.34	-191.26	-23.8			1.4				
998.65	-193.57	-23.9			0.9				
1002.70	-197.62	-25.1			0.3				
1005.56	-200.48	-24.1			1.0				
1011.28	-206.20	-24.5	68.6		1.2	5.56	0.12	0.40	12.31
1014.70	-209.62	-24.3			1.5				
1022.70	-217.62	-24.5			1.8				
1034.30	-229.22	-24.4			3.1				
1035.13	-230.05	-23.3			5.5				
1045.70	-240.62	-24.5			2.0				
1046.75	-241.67	-22.1			6.2				
1055.18	-250.10	-24.6			2.9				
1059.40	-254.32	-23.0			3.1				
1065.00	-259.92	-24.6			2.7				
1082.50	-277.42	-24.8			0.5				
1094.90	-289.82	-24.4			1.3				
1104.02	-298.94	-24.3			4.1				
1105.74	-300.66	-24.7			2.3				
1121.76	-316.68	-24.8			0.7				
1127.41	-322.33	-25.0			1.5				

Geochemical data, cont'd

Depth (m)	Depth (m from top of Bulli Coal seam)	δ^{13}Corg (‰ VPDB)	CIA	Kaolinite/ Illite	TOC (wt%)	Ni/Al (x10^4)	Mo/Al (x10^4)	U/Al (x10^4)	V/Al (x10^4)
1129.50	-324.42	-24.7			1.9				
1139.15	-334.07	-24.6			3.6				
1143.15	-338.07		81.1			8.28	0.15	0.00	18.18
1147.40	-342.32			0.4					
1151.00	-345.92	-23.5			1.6				
1162.40	-357.32	-23.4			1.7				
1167.40	-362.32	-22.0			3.1				
1172.47	-367.39	-23.5			0.9				
1192.42	-387.34	-23.2			1.7				
1209.77	-404.69	-24.1			0.8				
1217.25	-412.17	-23.9			1.1				
1220.30	-415.22	-23.8			1.7				
1223.69	-418.61	-24.0			0.9				
1230.05	-424.97	-24.0			1.1				
1231.82	-426.74	-23.7			1.0				
1239.00	-433.92	-23.7			1.3				
1241.70	-436.62	-24.2			1.5				
1246.38	-441.30	-23.8			1.1				
1251.00	-445.92	-23.3			0.7				

Supplementary References

1. Ludwig, K.R. *User's Manual for Isoplot 3.00*. (Berkeley Geochronology Center, 2003).
2. Balme, B.E. & Hennelly, J.P.F., 1956. Trilete sporomorphs from Australian Permian sediments. *Austr. J. Bot.* **4**, 240–260 (1956).
3. Balme, B.E. & Hennelly, J.P.F. Bisaccate sporomorphs from Australian Permian coals. *Austr. J. Bot.* **3**, 89–98 (1955).
4. Balme, B.E., Playford, G. Late Permian plant microfossils from the Prince Charles Mountains, Antarctica. *Rev. Micropaléo.* **10**, 179–192 (1967).
5. Mattinson, J.M. Zircon U-Pb chemical abrasion ("CA-TIMS") method: combined annealing and multi-step partial dissolution analysis for improved precision and accuracy of zircon ages. *Chem. Geol.* **220**, 47–66 (2005).
6. Schmitz, M.D. & Schoene, B. Derivation of isotope ratios, errors and error correlations for U-Pb geochronology using ^{205}Pb-^{235}U-(^{233}U)-spiked isotope dilution thermal ionization mass spectrometric data. *Geochem. Geophys. Geosys.* **8**, Q08006, doi:10.1029/2006GC001492 (2007).
7. Crowley, J.L., Schoene, B. & Bowring, S.A. U-Pb dating of zircon in the Bishop Tuff at the millennial scale. *Geol.* **35**, 1123–1126 (2007).
8. Jaffey, A.H. et al. Precision measurements of half-lives and specific activities of ^{235}U and ^{238}U, *Phys. Rev. C* **4**, 1889–1906 (1971).
9. Helby, R. Review of Late Permian and Triassic palynology of New South Wales. *Geol. Soc. Austr. Spec. Pub.* **4**, 141–155 (1973).
10. de Jersey, N.J. Palynology of the Permian-Triassic transition in the western Bowen Basin. *Geol. Surv. Qld. Pubs.* **374**, 1–39 (1979).
11. de Jersey, N.J. & Raine, J.I., 1990. Triassic and earliest Jurassic miospores from the Murihiku Supergroup, New Zealand. New Zeal. Geol. Surv. Paleo. Bull. **62**, 164 pp (1990).
12. de Jersey, N.J. & Hamilton, M. Triassic spores and pollen grains from the Moolayember Formation. *Geol. Surv. Qld. Pubs.* **336**, 1–61 (1967).
13. Foster, C.B. Permian plant microfossils of the Blair Athol coal measures, Baralaba coal measures, and basal Rewan Formation of Queensland. *Geol. Surv. Qld. Pubs.* **372**, 1–244 (1979).
14. Balme, B.E. in *Stratigraphic Boundary Problems: Permian and Triassic of West Pakistan: Palynology of Permian and Triassic strata in the Salt Range and Surghar Range, West Pakistan* (eds. Kummel, B. and Teichert, C.), 305–453 (Univ. Kansas Spec. Pubs., 1970).
15. Wilson, L.R. Permian plant microfossils from the Flowerpot Formation, Greer County, Oklahoma. *Circ. Ok. Geol. Surv.* **49**, 1–50 (1962).
16. de Jersey, N.J. Early Triassic miospores from the Rewan Formation. *Geol. Surv. Qld. Pubs.* **345**, 1–29 (1970).
17. Goubin, N. Description et répartition des principaux pollenites Permiens, Triasiques et Jurassiques des sondages du Bassin de Morondava (Madagascar). *Rev. L'Inst. Fr. Pétr.* **20**, 1415–1458 (1965).
18. de Jersey, N.J. Triassic miospores from the Esk Beds. *Geol. Surv. Qld. Pubs.* **357**, 40 pp (1972).
